# Progress of Surgery

**Published:** 1875-04

**Authors:** Jno. E. Owens

**Affiliations:** Lecturer on Surgery, Rush Medical College, Chicago


### Progress in Medical Sciences.

ARTICLE I.

### PROGRESS OF SURGERY.

BY JNO. E. OWENS, M.D.,

LECTURER ON SURGERY, RUSH MEDICAL COLLEGE, CHICAGO.

1. Hæmorrhoids. By Mr. W. COLLES. (*Dublin Journal of Medical Science*, June, 1874; *Braithwaite's Retrospect*, Jan., 1875.)

2. Iodide of Potassium in the System. By Dr. T. L. BRUNTON. (*Practitioner*, London, June, 1874; *Braithwaite's Retrospect*, Jan., 1875.)

3. On the Surgical Treatment of Gluteal Aneurism. By TIMOTHY HOLMES, Esq. (*London Lancet*, July 11 and 18, 1874; *Braithwaite's Retrospect*, Jan., 1875.)

4. On the Surgical Treatment of Inguinal and Femoral Aneurism. By TIMOTHY HOLMES, Esq. (*Lancet*, Oct., 1874; *Med. Times & Gazette*, July, 1874; *Braithwaite's Retrospect*, Jan., 1875.)

5. A Case of Death from the Administration of Bichloride of Methylene. (*British Med. Journal; The Boston Med. & Surgical Journal*, Jan. 21, 1875.)

1. Internal hæmorrhoids are vascular growths, resembling a nævus in children, or erectile tissue in adults. They may be entirely destroyed by injecting into each hæmorrhoidal tumor about twenty minims of the ordinary tincture of iron, by means of a hypodermic syringe. To enable this to be done, the piles must be forced down, so as to be extruded. In a case thus treated, on examination four weeks afterwards, no trace of the piles could be discovered, except three nodules, each the size of a shriveled currant.

2. When iodide of potassium is swallowed, it is absorbed from the stomach, passes in the blood to the salivary glands, and is excreted by them much more readily than by the kidneys. It is, however, again and again absorbed and excreted, and does not get out of the system unless a purgative is given to cause its expulsion from the bowels, before it can be reabsorbed. The same, says Dr. Brunton, is the case with the other iodides, such as those of lead and iron.

3. In the treatment of gluteal aneurism, present experience, says Dr. Holmes, points to the following conclusions:

*a.* Gluteal aneurisms, both traumatic and spontaneous, are very favorably circumstanced for the treatment by either rapid or gradual compression applied to the aorta or common iliac.

*b.* If this treatment does not succeed by itself, it may be supplemented by coagulating injection, or galvano-puncture, performed while the patient is narcotized, and the circulation commanded.

*c.* When such treatment fails, and particularly in aneurisms with imperfect or ruptured sacs, where it is not indicated, the internal iliac must be tied, when the surgeon thinks that he cannot find the artery outside the pelvis. But when the artery is accessible, the old operation, or the operation of Anel (the ligature of the affected artery as it approaches the tumor), should be practiced, according to the size and extent of the tumor.

*d.* The ligature of the internal iliac artery is liable to failure, in cases of spontaneous aneurism, from a diseased condition of the coats of the artery, and should always be avoided when other means of treatment are available.

4. On a review of the whole subject of femoral aneurism by the light of present experience, Prof. Holmes is led to the following conclusions:

*a.* That the operation of ligature of the external iliac artery has been, on the whole, fairly successful, as evidenced by a very small mortality in uncomplicated cases of hæmorrhage, and a mortality of about one-fourth in published cases of aneurism—a conclusion supported by the unpublished records of hospital practice, though there have been a few cases of recurrence of the aneurism.

*b.* That the operation on the superficial femoral, for aneurism situated in Hunter's canal, is a very successful operation.

*c.* That the ligature of the common femoral is a perfectly justifiable operation; though whether more or less trustworthy than that of the external iliac artery, we are not as yet in a position to judge.

*d.* That ruptured aneurism in the thigh has been treated with a large amount of success by the old operation (incision into the tumor, rapid removal of clots, ligature placed above and below the aneurism.)

*e.* That the ilio-femoral and femoral aneurisms have been treated with a very fair proportion of cures in the few instances on record, by rapid compression, applied to the aorta or common iliac, but that there is no evidence to show that this treatment is less dangerous or more successful than the operation on the external iliac artery, when the latter is feasible.

*f.* That compression, especially digital pressure, has been applied to the treatment of inguinal and femoral aneurism with striking success, though in what proportion of cases we do not as yet know. That the comparative ill-success of this method, in our hospital practice, is more calculated to raise doubts of the efficiency of the application than of the soundness of the method itself.

*g.* That in rare cases, direct pressure, or even manipulation, may be advantageous.*

*h.* The arterio-venous femoral aneurism should be treated by double compression, applied to the vein and artery; which failing, Mr. Spence's method of tying the artery above and below is the most hopeful measure; and when this is impracticable, either the old operation should be preferred, or the case abandoned.

*i.* That spontaneous aneurisms of the profunda have been diagnosed, and successfully treated by compression.

* The object of "manipulation" is to alter the relations of the layers of fibrin in the aneurismal sac, in order to bring about a deposition of fibrin which becomes entangled in the projecting surfaces of the displaced laminæ.

*j*. That recent traumatic aneurisms of branches of the external iliac or femoral, are best treated as wounds of these vessels—*i.e.*, either by compression or by ligature at the wounded part.*

5. The patient was suffering from caries of bone, near the lachrymal sac. Operation was determined on, and Mr. Buller administered bichloride of methylene, by means of a perforated leather inhaler, covered with flannel. Three drachms were inhaled, and in two minutes the breathing became stertorous. After the operation, the pulse at the wrist suddenly parted, and then the respiration ceased. All known means for restoring animation were employed.

* See Mr. Holmes' conclusions as to the treatment of popliteal aneurism, (*Chicago Med. Journal*, Jan., 1875, p. 42.)

